# Insights to maternal regulation of the paternal genome in mammalian livestock embryos: A mini-review

**DOI:** 10.3389/fgene.2022.909804

**Published:** 2022-08-19

**Authors:** Bradford W. Daigneault

**Affiliations:** Department of Animal Sciences, University of Florida, Gainesville, FL, United States

**Keywords:** paternal, embryo, imprint, allele, epigenomic, chromatin, environment

## Abstract

This mini-review focuses on current knowledge regarding maternal regulation of the paternal genome in early embryos of mammalian livestock species. Emphasis has been placed on regulatory events described for maternally imprinted genes and further highlights transcriptional regulation of the post-fertilization paternal genome by maternal factors. Specifically, the included content aims to summarize genomic and epigenomic contributions of paternally expressed genes, their regulation by the maternal embryo environment, and chromatin structure that are indispensable for early embryo development. The accumulation of current knowledge will summarize conserved allelic function among species to include molecular and genomic studies across large domestic animals and humans with reference to founding experimental animal models.

## Introduction

Mammalian spermatozoa contain highly compacted chromatin, thus providing stability and protection from environmental influence during sperm transport within the female reproductive tract. The completion of spermatogenesis results in protamination of sperm chromatin to replace histones, resulting in tightly compacted and condensed chromatin within the nucleus of the mature spermatozoa (Ribas-Maynou et al., 2021). Genomic integrity is also maintained by the ultrastructure of sperm, comprised of a plasma membrane and acrosome that remain largely intact until gamete interaction (Ward, 2010). Unassisted fertilization requires transit through the male vascular reproductive tract and the female uterus with exposure to seminal, vaginal, and uterine fluids that influence sperm function. Prior to gamete interaction, approximately 2–15% of sperm chromatin remains open and thus accessible to external environmental modulation (Ward, 2010). Current knowledge suggests that protaminated sperm chromatin is a non-randomized physiological event that completes at nuclear maturation during spermatogenesis (Ward and Coffey, 1991; Brunner et al., 2014). Species differences in sperm protamination that may alter susceptibility to DNA damage and the resilience of sperm DNA to decondensation can in part be attributed to two different types of protamines encoded in the genome (protamine 1 and protamine 2 family). Protamine 1 is conserved across all species, but the protamine 2 family is restricted to humans, mice, and horses and appears to be dependent upon protamine 1 for condensing sperm chromatin (Ribas-Maynou et al., 2021). Evidence suggests that genes on sperm euchromatin may contribute to minor embryonic genome activation (EGA), or at the least, are instrumental in the very early stages of embryo development prior to EGA (Hammoud et al., 2009; Brunner et al., 2014; Jung et al., 2017).

Unique genomic and epigenomic phenotypes of mature spermatozoa can include transgenerational inheritance stemming from environmentally induced modifications of the epigenome (Champroux et al., 2018; Donkin and Barres, 2018). Following fertilization, the paternal genome is tightly regulated by maternal stores of embryonic enzymes and methyltransferases in both a species-specific and parent-of-origin manner that are permissive and restrictive to monoallelic expression (Park et al., 2007; Richard Albert et al., 2020). Monoallelic expression refers to genes that are expressed exclusively from a single allele after fertilization. Monoallelically expressed genes fall into two categories. The first category describes genes that can be expressed randomly from a single allele, such as those usually observed during X-chromosome inactivation. The second category refers to those genes that are imprinted in a parent-of-origin manner, such that the gene is always expressed from the same allele (maternal or paternal) in every cell. Genomic imprinting is an example of monoallelic expression that occurs in the same cell when one of the two parental alleles is repressed by epigenetic modifications originating in the germline and is thus inactive (Chess, 2013).

Genomic imprinting is initially established in the germline during gametogenesis and is regulated by allele-specific DNA methylation at regions known as ICRs (imprinting control regions). Imprinting is an essential epigenetic mechanism that regulates monoallelic expression with the addition of a methyl group to the cytosine of genomic DNA, thereby preventing transcription stemming from the inability of transcription factors to access DNA and bind promoters (Jacob and Moley, 2005; Yagi et al., 2019). The inheritance of epigenetic modifications is influenced by reprogramming, resulting in the erasure and remodeling of epigenetic marks. Two distinct waves of DNA methylation reprogramming occur. First, primordial germ cells (PGCs) initially undergo DNA demethylation known as erasure, followed by remethylation of imprinted genes during gametogenesis through *de novo* methylation established in the germline at ICRs that remain stable in somatic cells post-fertilization, thus maintaining parent-of-origin expression (Ferguson-Smith, 2011; Yagi et al., 2019). The second wave of DNA methylation occurs after fertilization, when methylation marks inherited from gametes are erased again, except for those at imprinted loci. The paternal genome predominantly undergoes active demethylation, and the maternal genome is subject to passive demethylation, although exceptions may occur at specific regions (Zeng and Chen, 2019). Active demethylation of the paternal genome during embryo development must be accomplished for the establishment of the pluripotent epiblast to direct embryonic cell lineage specification and is regulated by maternal embryonic factors (Messerschmidt et al., 2014). Such regulation of the paternal genome by the maternal environment clearly illustrates the influence of oocyte quality and follicular dynamics on embryo developmental potential that are independently susceptible to environmental influence during sensitive windows of oocyte maturation.

Decades of research using mouse models have demonstrated that the paternal genome has a major influence on placental development (Barton et al., 1984; McGrath and Solter, 1984). More recent equine models also show enrichment for monoallelic expression of paternally derived genes in trophoblast tissue, suggesting an important role in placental function (Wang et al., 2013; Dini et al., 2021). The combination of open chromatin and reliance on monoallelic expression for trophoblast function emphasizes the importance of genomic and epigenomic stability of the paternal genome. Global concern for gene-environment interactions both pre- and post-fertilization lends susceptibility to impact on sperm function and embryo development. Disruption or destabilization of genomic integrity in either scenario is likely to alter developmental potential. Sperm transit through the female reproductive tract serves as an additional route whereby the internal milieu may also impact the integrity of the paternal genome, thereby modulating monoallelic expression post-fertilization. The increased awareness of paternal contributions to early embryo development ultimately requires further understanding of maternal regulation of the sperm genome post-fertilization (Daigneault, 2021). This mini-review summarizes current knowledge concerning maternal regulation of the paternal genome in early mammalian livestock embryos and methods of regulating gene expression that include epigenetic mechanisms with species distinctions.

## Embryonic interactions with maternally imprinted genes

Monoallelic expression in early embryo development is confounded by species differences that are temporal, restricted to cell lineage, or tissue-specific. In mammals, 263 imprinted genes have been identified, and species specificity reveals some conservancy (Duan et al., 2018). High-throughput sequencing of the ovine fetus has recently expanded the number of known imprinted genes to 34 (Duan et al., 2018). Although the regulation of imprinted genes may be mostly conserved across species, differences in the timing of embryo development are likely to influence expression as observed in developmental control genes such as *OCT4*, which is restricted to inner-cell mass (ICM) lineages in mice but ubiquitous throughout trophectoderm and the ICM of cattle and human embryos (Frum et al., 2013; Fogarty et al., 2017; Daigneault et al., 2018). Insulin-like growth factor 2 (*IGF2*) is among the most well-described paternally expressed/maternally imprinted genes. Early developmental roles of IGF2 as a growth hormone appear regulated by the expression of the maternal growth suppressor PHLDA2*,* demonstrating the complex yet crucial role of maternal interaction with a paternally expressed growth factor (Demetriou et al., 2014). The IGF2–PHLDA2 interaction perhaps exemplifies the “parental conflict hypothesis,” whereby paternal influence on fetal growth is achieved through silencing of the maternal allele while maternal influences limit expression, thereby reserving resources for littermates and maternal health (Saldivar Lemus et al., 2017). More classic representation of this phenom includes paternally expressed *IGF2* and the maternally expressed receptor *IGF2R* (insulin-like growth factor 2 receptor)*,* where transgenic loss-of-function in mice demonstrates a reduction and increase in birth weight, respectively (DeChiara et al., 1990; Wang et al., 1994). In humans, growth disorders attributed to *IGF2* include Beckwith–Wiedemann (overgrowth) and Silver–Russel (growth restricting) syndromes (Bergman et al., 2013). Of the 50 known imprinted genes in cattle, more than half are paternally expressed (www.geneimprint.com). Bovine *MKRN3*, *MAGEL2*, and *NDN* are three protein-coding maternally imprinted genes that are regulated by DNA methylation in placental tissues controlled by the PWS-DMR (Prader–Willi syndrome—differentially methylated region) located on chromosome 21 (human chromosome 15) in the promoter of the *SNRPN* gene (Li et al., 2021). Loss of function from these genes in humans may lead to Prader–Willi syndrome in humans, but less is known regarding requirements for early embryo development and placental function in non-rodent models.

### Control of imprinted genes

Aberrant methylation patterns at DMRs of genes such as *IGF2*, *H19*, and *GTL2* in Y chromosome-containing prospermatagonia during spermatogenesis have been explored as specific epigenetic alterations to maternally imprinted genes of infertile men (Boissonnas et al., 2010). Proper regulation of paternal imprinting by the embryo environment is critical for preventing ectopic gene expression during early cell lineage specification where pluripotency can concomitantly be achieved (Park et al., 2007). Transcriptional activity of IGF2 is regulated by H19, a non-coding RNA that is imprinted in humans, rodents, and cattle (Smith et al., 2007). *H19* is paternally imprinted and therefore expressed only from the maternal allele, further demonstrating maternal regulation of a paternally derived monoallelic gene. Loss or aberrant paternal imprinting of *H19* results in fetal growth restriction, or Silver–Russel syndrome (Soejima and Higashimoto, 2013). Loss of methylation at DMRs of both *IGF2* and *H19* at variable CpG positions is correlated with an increase in abnormal sperm and infertility in men (Boissonnas et al., 2010). In either case, epigenetic modification or dysregulation in a parent-of-origin-specific manner remains detrimental to early embryo development.

Further examples of imprinted genes associated with male infertility include *GNAS* and the tumor suppressor *DIRAS3*, although the disruption of methylation patterns in correlation with fertility remains unclear (Tang et al., 2018). Whole-genome bisulfite sequencing (WGBS) and RNA-sequencing approaches in porcine embryos facilitate the identification of imprinted genes, where *DIRAS3* is maternally imprinted in the embryo and remains hypermethylated in other tissues including the hypothalamus (Ahn et al., 2021). The regulation of *DIRAS3* and other imprinted genes in the early embryo has not been fully elucidated, and comprehensive mechanisms are beyond the scope of this review.

## Parental chromatin dynamics

Chromatin dynamics of the paternal genome are also regulated by maternal stores of proteins and protein complexes but do not appear to be conserved from rodents to large animals. Asymmetric assembly of paternal and maternal chromatins is acquired at the gamete level and requires rearrangement post-fertilization (Burton and Torres-Padilla, 2014). The protamine-compacted sperm genome is exchanged by maternal histones to form constitutive heterochromatin (cHC). Modified histones of human paternal cHC appear to be retained upon delivery to the oocyte and are recognized in the zygote by the H3K9/HP1 pathway of maternal chromatin modifiers where they are bound by maternal HP1 (van de Werken et al., 2014). Remarkably, the paternal histone modifications are retained during mitotic cleavage of the embryo to form a paternal, intergenerational, epigenetically inherited signature of functional cHC in human embryos (van de Werken et al., 2014). A different regulation exists for mouse embryos by which the paternal cHC canonical modifications have not been described but are established in the embryo by a different source of maternal proteins (Polycomb) (Santos et al., 2005; Puschendorf et al., 2008). Such findings exemplify the dynamics of maternal regulation of paternal chromatin while demonstrating the species specificity of evolutionary divergent pathways.

### Epigenetic regulation of paternal transcripts

The maternal transcriptome of the mammalian embryo has evolved unique methods of regulating paternally expressed genes through epigenetic modifications to the paternal genome. These epigenetic regulatory processes appear species specific and are rather unique due to their indirect methods of preventing expression as a transcriptional repression mechanism through hypermethylation. *De novo* DNA methylation (DNAme) during spermatogenesis results in a densely methylated sperm genome except for CpG islands (CGIs) that are predominately hypomethylated in mature sperm (Richard Albert et al., 2020). In mice and cattle, active demethylation occurs rapidly after fertilization and is male pronucleus specific (Park et al., 2007). DNA 5-hydroxymethylcytosine (5hmC) catalyzation by ten-eleven translocation (TET) protein is important for DNA methylation reprogramming in bovine embryos. TET proteins were initially characterized as enzymes involved in the oxidation of 5mC to 5hmC (Tahiliani et al., 2009). Demethylation of the parental genome is disparate post-fertilization (Inoue and Zhang, 2011; Iqbal et al., 2011; Wossidlo et al., 2011), where TET3 actively converts 5mC to 5hmC in the paternal pronucleus while the maternal pronucleus remains protected from TET3 (Nakamura et al., 2007; Nakamura et al., 2012; Bakhtari and Ross, 2014).

Active demethylation of the paternal pronuclei may be conserved, but limited reports exist in other species such as sheep (Hou et al., 2008; Masala et al., 2017). Interspecies comparison of DNA methylation dynamics in the preimplantation embryo revealed high levels of TET3 in porcine embryos, indicating similar active demethylation dynamics of the paternal genome (Ivanova et al., 2020). Remethylation following global embryonic demethylation is critical for the erasure of undesirable epigenetic modifications (epimutations) (Park et al., 2007). *De novo* methylation of the paternal chromatin *via* maternal embryonic DNMT3A restricts the expression of paternal transcripts that are precociously activated in *Dnmt3a*-null mouse embryos. These findings illustrate dual roles for *DNMT3A* that include maternal imprinting and methylation of a distinct population of genes on the paternal genome by initial embryo cleavage to inhibit expression in the preimplantation embryo for epigenetic reprogramming and transcriptional silencing of the paternal genome (Richard Albert et al., 2020).

The precedence for epigenetic pathways of maternal origin that control the regulation of paternal contributions to early embryo development has been exemplified in plants, insects, and lower mammalian model species as evolutionary divergent (Autran et al., 2011). *DPPA3* (PGC7, Stella) is an additional example of a maternal factor that asymmetrically remodels paternal and maternal bovine pronuclei and also drives ICM cell numbers in preimplantation embryos (Bakhtari and Ross, 2014). The pattern of *DPPA3* expression in embryos is consistent with other maternal effect genes (MEGs), which typically encode RNAs that are required for early embryonic development. Thus, the expression of MEGs is often more abundant in oocytes and then decreases until EGA. Knockdown of *DPPA3* in bovine oocytes suggests a protective mechanism for the maternal pronucleus from 5mC oxidation to 5hmC as demonstrated by increased levels of 5hmC in *DPPA3* knockdown embryos. In effect, DPPA3 may be described as a maternal transcript that regulates paternal zygotic gene expression by modulation of the epigenome in a parent-specific pattern. In humans, DPPA3 can inhibit TET2 and TET3 activities by directly binding to the catalytic domain of these enzymes. Like DPPA3, GSE (gonad-specific expression gene) preferentially binds to the paternal chromatin at the pronuclear stage and plays a role in the maintenance of 5mC as demonstrated by 5hmC accumulation in GSE knockdown mouse embryos (Hatanaka et al., 2013). Combined, these maternal factors exemplify interactions that regulate the expression of the male genome post-fertilization in a spatio-temporal and species-dependent manner.

## Conclusion

The growth of -omics technologies in the 21st century, coupled with gene editing strategies, increased awareness of male fertility, and environmental factors that contribute to reproductive deficiencies has maneuvered most livestock industries toward genomics-driven approaches to reproduction that require a unique marriage to research in basic sciences. Advances to describe the regulation of gene expression and unique epigenetic modifications to the genome include key differences in parent-of-origin expression that appear species specific in many cases. Modulation of the paternal genome is partially regulated by the maternal environment and dependent upon protein, methyltransferases, and non-coding RNAs of maternal origin. The complexity of maternal regulation of the paternal genome requires further understanding of epigenetic mechanisms that may be inherent to the embryo or modulated by a dynamic and plastic environment stemming from oocyte development through the preimplantation embryo. Clear emphasis on research that addresses trophectoderm function to mitigate pregnancy loss has further illustrated the role of maternally imprinted genes in livestock embryos. The maternal regulation of paternally expressed monoallelic genes will ultimately influence reproductive efficiencies in livestock and other large animals where concern for gene–environment interactions continues to develop as an emergent field required for global sustainability in food production with high relevance to *in vitro* embryo production.
